# Factors associated with the availability and affordability of essential cardiovascular disease medicines in low- and middle-income countries: A systematic review

**DOI:** 10.1371/journal.pgph.0000072

**Published:** 2022-03-23

**Authors:** Ali Lotfizadeh, Benjamin Palafox, Armin Takallou, Dina Balabanova, Martin McKee, Adrianna Murphy

**Affiliations:** 1 PASHA, Los Angeles, California, United States of America; 2 Department of Health Services Research and Policy, Centre for Global Chronic Conditions, London School of Hygiene and Tropical Medicine, London, United Kingdom; 3 School of Medicine, Oregon Health and Science University, Portland, Oregon, United States of America; 4 Department of Global Health and Development, Centre for Global Chronic Conditions, London School of Hygiene and Tropical Medicine, London, United Kingdom; Bangladesh University of Health Sciences, BANGLADESH

## Abstract

Despite their potential to prevent or delay the onset and progression of cardiovascular disease (CVD), medicines for CVD remain unavailable and unaffordable to many in low- and middle-income countries (LMICs). We systematically reviewed the literature to identify factors associated with availability and affordability of CVD medicines in LMICs. A protocol for this study was registered on the PROSPERO register of systematic reviews (CRD42019135393). We searched Medline, EMBASE, Global Health, Cumulative Index to Nursing and Allied Health Literature, EconLit, Social Policy and Practice, and Africa Wide Information for studies analyzing factors associated with the presence of medicines (availability) or the price of these medicines as it relates to ability to pay (affordability) in LMICs. We performed a narrative synthesis of the results using an access to medicines framework that examines influences at different levels of the health system. We did not conduct a meta-analysis because of the differences in analytic approaches and outcome measures in different studies. The search was conducted in accordance with PRISMA guidelines. Of 43 studies meeting inclusion criteria, 41 were cross-sectional. Availability and affordability were defined and measured in different ways. A range of factors such as sociodemographic characteristics, facility tier, presence of medicines on national essential medicine lists, and international subsidy programs were examined. The studies had variable quality and findings were often inconsistent. We find gaps in the literature on factors associated with availability and affordability of CVD medicines, particularly at the health program level. We conclude that there is a need for experimental and quasi-experimental studies that could identify causal factors and effective responses. Such studies would help further our understanding of how complex multifactorial influences impact these outcomes, which could inform policy decisions. Along with this, greater standardization of definitions and measurement approaches of availability and affordability are needed to allow for more effective comparisons.

## Introduction

Control of blood pressure and lipid levels by pharmacotherapy is a core element of primary and secondary prevention of cardiovascular disease (CVD). Antihypertensive drugs are known to reduce the incidence of cardiac and cerebrovascular diseases [1], as are statins [2] and antiplatelet agents [3]. Despite the known efficacy and cost-effectiveness of these medicines [4], their use remains far from optimal, especially in low- and middle-income countries (LMICs), where 80% percent of CVD-related mortality occurs [5]. The World Health Organization (WHO) has advocated for a target goal of 50% of eligible people receiving medicines for CVD [6]. Yet, in LMICs, fewer than 30% with hypertension receive treatment and less than 8% achieve blood pressure control [7]. In one study of only low-income countries, fewer than 10% of those with a history of coronary heart disease or ischemic stroke received treatment with antiplatelet drugs or lipid-lowering agents, contrary to international guidelines [8].

One reason for this treatment gap is poor access to medicines. Access has multiple dimensions but two that are commonly explored are availability and affordability. A recommended definition of availability compares the quantity of a medicine required in relation to its presence at health facilities. Affordability is captured by a medicine’s price relative to an individual or household’s ability to pay [9]. Research in LMICs shows that the availability and affordability of CVD drugs are associated with greater odds of patients using them and with a lower risk of adverse cardiovascular outcomes [10].

Although the WHO recommends at least 80% medicine availability for CVD [6], this target is not being met in LMICs. Many patients with hypertension require combination therapy but only 13% of communities in low-income countries live in areas where all four main classes of antihypertensives are available and only 30% of households can afford them [11]. While many studies describe the scale of the problem, fewer seek to explain the multitude of factors involved and their interrelationships. Our objective is to systematically review the literature to identify such factors in LMIC to inform appropriate policy responses and future research.

## Materials and methods

We conducted a systematic review of the literature on factors associated with availability and affordability of medicines for CVD in LMICs. The protocol for this review was registered and published on the PROSPERO register of systematic reviews (CRD42019135393). Findings are reported according to the PRISMA guidelines.

### Inclusion and exclusion criteria

We included published studies in any language that reported original data *and* analyzed interventions or factors associated with availability and/or affordability of CVD medicines in countries defined as low- or middle-income according to the 2021 World Bank classification. We included quantitative, qualitative, or mixed-method comparative studies, using experimental, quasi-experimental, or observational designs. We included any measure of availability providing information on the physical presence of medicines at health facilities or home. For affordability, we included studies that considered the price of a medicine incurred by an individual or household in relation to their ability to pay [12]. Studies that only provided information on the price of medicines were excluded. Studies that examined an individual or household’s ability to obtain medicines for free (as opposed to having to pay for medicines) were included, because free medicines are, by definition, more affordable than medicines at any price [13]. We included studies that directly measured availability and/or affordability, those that used information from other surveys, and those that asked respondents to report on availability and/or affordability.

Studies with data from more than one country were included if the majority were LMICs. We focused on CVD medicines in the following three categories: antihypertensive agents, platelet aggregation inhibitors, and lipid-lowering agents. Studies that reported data on a basket of medicines were included if CVD medicines were a part of that basket.

#### Search strategy

We searched MEDLINE, EMBASE, Global Health, Cumulative Index to Nursing and Allied Health Literature, EconLit, Social Policy and Practice, and Africa Wide Information for studies in any language published after the year 2000. We also conducted a grey literature search using the website of the Institute of Development Studies, the WHO Repository, the World Bank Repository, and Google. We restricted our search to publications from the year 2000 onward because of a paucity of health systems literature prior to this date. We performed our initial search in June 2020 and updated the search in July 2021. The references of all included records were manually reviewed. The search strategy was developed in collaboration with a librarian at the London School of Hygiene and Tropical Medicine with expertise in systematic review methodology. The search terms were subsequently peer-reviewed by another information specialist at the London School of Hygiene and Tropical Medicine not involved in developing the search strategy. More details on the search strategy are presented in Table 1 and the complete search terms can be found in Appendix 1 (S1 Table). Non-communicable disease (NCD) medicines were included in our search because an initial scoping review revealed papers that examined availability and/or affordability of a basket of NCD medicines, including CVD drugs.

**Table 1:** Search strategy combining three themes: Medicines for NCDs and CVD, availability or affordability, and LMICs.

Main Concept	Components of Main Concept	Sample Terms from Medline Search
	[Terms for CVD Medicines]	[cardiovascular agents/ OR antihypertensive agents/ OR hypolipidemic agents/]
Medicines for NCDs and CVD	**OR** [(Terms for CVD) AND (Terms for Medicines)]	[(cardiovascular diseases/ OR exp hypertension/ OR exp hyperlipidemias/ OR cardiovascular disease*.ti,ab.) AND (exp pharmaceutical preparations/ OR medication*.ti,ab.)]
**AND**	**OR** [(Terms for NCD) AND (Terms for Medicines)]	[(exp chronic disease/ OR chronic disease*.ti,ab. OR chronic condition*.ti,ab. OR NCD.ti,ab.) AND (exp pharmaceutical preparations/ OR medication*.ti,ab.)]
Availability/Affordability **AND**	[Terms for Availability OR Affordability]	[availab*.ti,ab. OR supply.ti,ab. OR drug costs/ OR exp fees, pharmaceutical/ OR affordab*.ti,ab.]
LMIC	[Terms for LMIC OR List of LMIC]	[developing countries/ OR exp africa south of the sahara/ OR Armenia/ OR Armenia.ti,ab.]

The results were screened independently by two reviewers at the title/abstract level and studies not meeting inclusion criteria were excluded. Both reviewers subsequently screened full texts of retained articles independently, excluding those that did not meet inclusion criteria. Discrepancies were discussed with a third reviewer and a consensus was reached.

### Data extraction

The following information was extracted from all studies: language, study implementation year, country, study design, study setting, sample size, survey method used, factors analyzed, methodology, medicines studied, outcome measure, definition of availability and/or affordability, how availability and/or affordability was quantified, and study findings including the statistical parameters used (e.g. odds ratios, proportions, chi-squared values, p-values, and confidence intervals). Studies that were not in English were translated using Google Translate. Where translations were not clear, we used a dictionary and consulted colleagues with knowledge of the language.

### Risk of bias assessment

We assessed quality for observational studies using a method previously published by Maimaris [14], which examines three domains: selection bias, information bias (differential and non-differential misclassification), and confounding, thereby providing more precise information on studies than is done by some other instruments whose primary purpose is to determine whether to include a study or not. To evaluate non-differential misclassification, we assessed the reliability of the measures used for availability and affordability. The risk of bias tool for observational studies is presented in Appendix 2 (S2 Table). For randomized controlled trials, the revised Cochrane tool was used [15]. This tool measures bias in the following domains: randomization, timing of identification/recruitment in relation to timing of randomization, deviations in intended interventions, missing outcome data, outcome measurements, and selection of the reported results. Two reviewers independently performed risk of bias assessment and resolved discrepancies with discussion.

### Conceptual framework and narrative synthesis

We organized our findings according to a framework of health systems constraints adapted to access to medicines in LMICs [16]. This framework proposes different levels of the health system as follows: 1) individuals, households, and communities, 2) health service delivery, 3) health sector (or program), 4) national context (public policies cutting across sectors), 5) international context. We considered sociodemographic factors and geographic location at level 1. At level 2, we included characteristics of a health facility or service delivery arrangements at that facility. At the health sector level, we examined country or region-wide health programs or policies. At the national context, policies extending beyond the health sector were examined. At level 5, international programs or arrangements that were related to the availability or affordability of medicines were examined. We coded factors associated with availability and affordability into the different levels and performed a narrative synthesis of the data.

## Results

**Fig 1:**
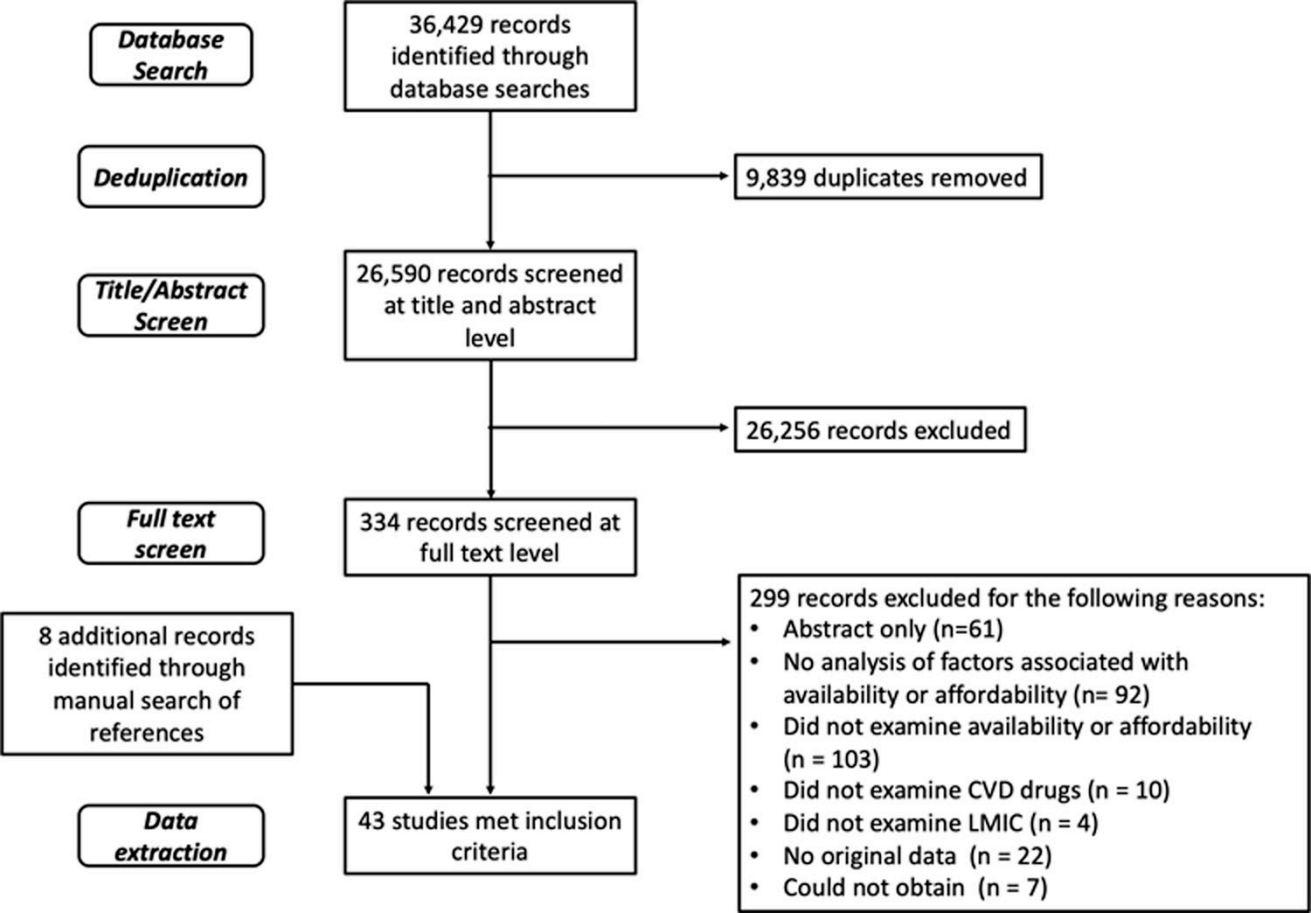
PRISMA flowchart. Flowchart of the selection and screening process of studies on availability and affordability of CVD medicines.

The results of the screening process are reported in the PRISMA flowchart (Fig 1). The initial search yielded 32,352 results and an additional 4,077 were retrieved from the updated search, resulting in 36,429 titles. Of these, 9,839 were duplicates, leaving 26,590 citations. Title and abstract screening resulted in 334 studies, of which 327 were retrieved for full-text screening. The remaining seven studies could not be accessed because they were in journals to which our library and no affiliated libraries subscribe. Thirty-five studies met inclusion criteria and an additional eight were included through a manual search of the references.

### Study characteristics

Of the included studies, 40 were in English [17–56], two were in Spanish [57, 58], and one was in Portuguese [59]. Thirty-four studies focused on availability [17, 18, 20–24, 26–36, 38, 40–42, 44, 45, 47, 48, 50–54, 56, 58, 59], seven on affordability [19, 25, 37, 39, 43, 46, 55], and two on both [49, 57]. Risk of bias assessment revealed heterogeneity in the quality of the included studies. Fifteen of the cross-sectional studies had one or more domains with high risk of bias [23, 24, 27, 32, 35, 37, 43, 49–55, 57] while another seven had unclear risk of bias in at least one domain [20, 22, 30, 44, 53, 54, 59]. Detailed characteristics of included studies and the results of risk of bias assessments are described in Table 2. Seven of the included studies used the WHO Health Action International survey [20, 22, 23, 26, 28, 30, 31, 53, 56] and six used the Service Availability and Readiness Assessment tool to measure medicine availability [17, 18, 24, 27, 29, 38, 52]. Definitions and quantification approaches used to measure availability and affordability are presented in Tables 3 and 4, respectively.

**Table 2:** Characteristics of identified studies.

Author Year Country	Study Design (Sample Size and Setting)	Medicines Studied	Key Findings	Risk of Bias
**Availability**				
Adinan [17] 2019 Tanzania	Cross-sectional (34 Facilities)	Antihypertensive agents	No significant difference in availability at different tier facilities	Low risk of bias in all domains
Albelbeisi [50] 2020 West Bank and Gaza	Cross-sectional (52 Facilities)	CVD Medicines	No significant difference in availability in different regions	High risk for confounding
Armstrong-Hough [18] 2018 Uganda	Cross-sectional (196 Facilities)	NCD medicines	Being a higher tier facility, being a private facility (versus public), having greater infrastructure, and integrating NCD care with HIV testing and counseling associated with significantly greater availability; providing HIV care associated with significantly lower availability	Low risk of bias in all domains
Bazargani [20] 2014 23 LMICs	Cross-sectional (2290 Facilities)	Basket of medicines for chronic and acute conditions	Significantly higher median availability of medicines on the national essential medicine list than those not on the list	Unclear risk for selection bias
Bintabara [51] 2020 Tanzania	Cross-sectional (770 Facilities)	CVD Medicines	Significantly greater availability at private facilities than at public facilities	High risk for confounding
Bintabara [21] 2018 Tanzania	Cross-sectional (725 Facilities)	Antihypertensive agents	Significantly greater odds of preparedness for hypertension services at private facilities, at higher tier facilities, and at urban facilities	Low risk of bias in all domains
Cameron [22] 2011 40 Countries	Cross-sectional (2779 Facilities)	Medicines for chronic conditions	Significantly higher mean availability at private facilities than public facilities	Unclear risk for selection bias
Cepuch [23] 2012 Kenya	Cross-sectional (56 Facilities)	NCD medicines CVD medicines	No significant difference in mean availability of NCD medicines at private mission and public facilities; significantly higher availability of hydrochlorothiazide at urban facilities but no significant differences for other CVD medicines	High risk for confounding
Duong [24] 2019 Vietnam	Cross-sectional (89 Facilities)	CVD medicines	Significantly lower availability in the mountainous region as compared to other regions of the country	High risk for confounding and selection bias
Ekenna [52] 2020 Nigeria	Cross-sectional (60 Facilities)	Basic essential medicines	Significantly greater availability at urban facilities than at rural ones	High risk for confounding
Fang [26] 2013 China	Cross-sectional (80 Facilities in First Survey, 144 Facilities in Second Survey)	Basic essential medicines	Significant decrease in availability after implementation of a policy of zero-mark-ups on drugs and introduction of a provincial essential medicine list	Low risk of bias in all domains
Ibrahim [53] 2020 Yemen	Cross-sectional (30 Facilities)	CVD Medicines	No significant different in availability in different cities, significantly greater availability at private facilities	Unclear risk for selection bias and high risk of confounding
Jigjidsuren [27] 2019 Mongolia	Cross-sectional (146 Facilities)	Basic essential medicines	No significant difference in availability between higher and lower tier facilities	High risk for confounding
Kasonde [28] 2019 Bangladesh	Cross-sectional (135 Facilities)	Basket of medicines for chronic and acute conditions	Significantly lower availability of medicines at public facilities compare to private facilities, no significant difference in availability of medicines on the national essential medicine list and those not on the list	Low risk of bias in all domains
Katende [29] 2015 Uganda	Cross-sectional (28 Facilities)	Antihypertensive agents	Significantly higher medicine availability at higher tier facilities	Low risk of bias in all domains
Khanal [30] 2019 Nepal	Cross-sectional (60 Facilities)	NCD Medicines	No significant difference in medicine availability in different geographic regions of the country	Unclear risk for selection bias
Kibridge [31] 2017 Uganda	Cross-sectional (155 Facilities)	CVD medicines	Significantly higher medicine availability at private facilities compared to public ones only for certain classes of CVD medicines	Low risk of bias in all domains
Mendes [59] 2014 Brazil	Cross-sectional (29,228 Facilities)	Basic essential medicines CVD medicines	Significantly higher odds of essential medicine availability at facilities with greater levels of infrastructure and at facilities dispensing psychotropic medicines; significantly higher availability of antihypertensives and cardiology drugs at higher tier health facilities	Unclear risk for selection bias
Minaei [32] 2019 Iran	Cross-sectional (60 Facilities)	Basic essential medicines	No significant difference in availability at public and private facilities or across difference cities	High risk for confounding
Musinguzi [33] 2015 Uganda	Cross-sectional (126 Facilities)	Antihypertensive agents	Significantly higher availability at higher tier facilities	Low risk of bias in all domains
Mutale [34] 2018 Zambia	Cross-sectional (46 Facilities)	CVD medicines	Significantly higher availability of calcium channel blockers at urban facilities but no significant difference for other CVD medicine classes	Low risk of bias in all domains
Oliveira [35] 2016 Brazil	Cross-sectional (12,725 Households)	Medicines for chronic conditions	Significantly higher availability at private facilities and at facilities adopting the Farmacia Popular Program^a^	High risk for information bias
Oyekale [36] 2017 Nigeria	Cross-sectional (2480 Facilities)	Basic essential medicines	Significantly higher availability at higher tier facilities and facilities with greater infrastructure; no significant difference in availability between urban and rural facilities or between private and public facilities	Low risk of bias in all domains
Peck [38] 2014 Tanzania	Cross-sectional (24 Facilities)	Antihypertensive agents	No significant difference in availability at different tier facilities	Low risk of bias in all domains
Resendez [58] 2000 Mexico	Cross-sectional (67 Facilities)	Antihypertensive agents	No significant difference in availability between urban and rural facilities and different tier facilities	Low risk of bias in all domains
Restinia [54] 2021 Indonesia	Cross-sectional (Unknown number of facilities)	Antihypertensive agents	Significant increase in the availability of amlodipine and lower dose captopril, a decrease in the availability of hydrochlorothiazide, and no change in availability of higher dose captopril or nifedipine after implementation of the National Health Insurance scheme which changed payments from fee for service to diagnosis-based payments and required drugs to be ordered from the national formulary	Unclear risk for selection bias, unclear risk for differential and non-differential misclassification, and high risk for confounding
Rockers [40] 2019 Kenya	Cluster Randomized Trial (571 Individuals, 127 Facilities)	Antihypertensive agents	Novartis access program providing medicines at $1 per treatment per month was associated with significantly greater odds of availability of amlodipine but not other medicines at health facilities; the intervention was not associated with significantly greater odds of availability of any medicines at home	Low risk of bias in all domains
Rockers [41] 2018 Kenya	Cross-sectional (639 Individuals)	Antihypertensive agents	Significantly higher likelihood of availability in higher socioeconomic households	Low risk of bias in all domains
Saeed [56] 2021 Pakistan	Cross-sectional (81 Facilities)	CVD Medicines	Significantly lower mean medicine availability at public facilities than at private facilities, significantly lower availability of generics than originator brands, significantly greater availability of medicines on the national essential medicine list	Low risk of bias in all domains
Su [42] 2017 China	Cross-sectional (3362 Facilities)	Antihypertensive agents	Significantly higher availability at higher tier health facilities and of medicines recommended by national guidelines	Low risk of bias in all domains
Uzuchukwo [44] 2012 Nigeria	Cross-sectional (33 Facilities)	Basic essential medicines	Significantly higher availability at facilities adopting a revolving drug fund	Unclear risk for confounding and information bias
Vialle-Valentin [45] 2015 5 LMICs	Cross-sectional (1867 Individuals)	Medicines for chronic conditions	No significant difference in odds of medicine availability at home based on household education level in any country; significantly greater odds of medicine availability in poorer households in Jordan and lower odds of medicine availability in poorer households in Kenya, no differences observed in other countries	Low risk of bias in all domains
Wirtz [47] 2018 Kenya	Cross-sectional (445 Individuals)	Antihypertensive agents	Significantly greater odds of medicine availability in households with higher education level and higher socioeconomic status; no significant difference in odds of availability between urban and rural facilities	Low risk of bias in all domains
Yang [48] 2015 China	Cross-sectional (90 Facilities)	Basic essential medicines	Significant positive association between being in a central province and medicine availability	Low risk of bias in all domains
**Affordability**				
Ashigbie [19] 2020 Kenya	Cross-sectional (137 Facilities)	Basket of medicines for chronic and acute conditions	Significantly greater likelihood of receiving medicines for free at public facilities than private facilities	Low risk of bias in all domains
Emmerick [25] 2020 Brazil	Retrospective interrupted time series (25,150 Facilities)	Antihypertensive agents	Significant reduction of out of pocket payment for medicines to zero after elimination of co-payments	Low risk of bias in all domains
Leao Tavares [43] 2016 Brazil	Cross-sectional (12,725 Individuals)	Medicines for chronic conditions	Significantly higher likelihood of obtaining medicines for free among individuals from lower socioeconomic backgrounds and those without health insurance	High risk for information bias
Paniz [37] 2010 Brazil	Cross-sectional (2460 Individuals)	Medicines for hypertension and diabetes	Significantly greater likelihood of obtaining medicines for free at health units providing more comprehensive NCD care with greater follow-up	High risk for information bias
Perlman [39] 2011 Russian Federation	Cross-sectional (4215 Households)	Basic essential medicines	Significantly greater likelihood of household-reported affordability of medicines among those from higher socioeconomic backgrounds; significantly greater likelihood of household reported affordability among men with compulsory health insurance in 1994 but not in 1998 or 2004	Low risk of bias in all domains
Restrepo 2020 Brazil	Cross-sectional (289 individuals)	Basic essential medicines	No difference in affordability based on sex, age, or socioeconomic status, significantly greater affordability for those with lower education, chronic disease, no supplementary health insurance plan, and those who primarily obtained medicines from the Sistema Unico de Saude^b^	High risk for confounding
Viana [46] 2015 Brazil	Cross-sectional (27,333 Individuals)	Medicines for chronic conditions	Significantly greater odds of obtaining medicines for free among those from lower socioeconomic backgrounds and those without health insurance	Low risk of bias in all domains
**Both**				
Contreras-Loya [57] 2013 Mexico	Cross-sectional (30 Facilities)	Basic essential medicines	No significant difference in availability of medicines at facilities with outsourced pharmacies and those operated by the state health service; significantly greater likelihood of obtaining medicines for free at outsourced pharmacies	High risk for confounding
Fernandopulle [49] 2019 Sri Lanka	Cross-sectional (1008 Individuals)	NCD medicines	No significant differences in availability or affordability of medicines reported by men or women	High risk for confounding and information bias

a Programs adopting the Farmacia Popular Program provide a wider range of medicines and charge a fee for some of them, their counterparts provide fewer medicines all at no cost

b The Sistema Unico de Saude is a program that aims to increase access to medicines by providing medicines for free

**Table 3:** Approaches to measuring availability by different studies.

Study	Availability Definition	Availability Quantification Approach	Verified through direct observation	Checked for medicine expiry date?
Adinan 2019 [17]	Physical presence of drug	Proportion of facilities where at least one of three classes of medicines was available	Yes	Yes
Albelbeisi 2020 [50]	Physical presence of drug	Proportion of facilities where medicine was available	Not stated	Not stated
Armstrong-Hough 2018 [18]	Physical presence of drug	Proportion of medicines available	Not stated	Not stated
Bazargani 2014 [20]	Physical presence of drug	Proportion of facilities where medicine was available	Not stated	Not stated
Bintabara 2020 [51]	Physical presence of drug	Proportion of facilities where at least one type of medicine was available	Yes	Not stated
Bintabara 2018 [21]	Physical presence of drug	Incorporated into preparedness score, where drug availability was counted as one of three equally weighted components of facility preparedness	Not stated	Not stated
Cameron 2011 [22]	Physical presence of drug	Proportion of facilities where medicine was available	Not stated	Not stated
Cepuch 2012 [23]	Physical presence of drug	Proportion of facilities where medicine was available	Not stated	Not stated
Contreras-Loya 2013 [57]	Physical presence of drug	Proportion of medicines available; proportion of facilities where all medicines from a predetermined list were available	Not stated	Not stated
Duong 2019 [24]	Not stated	Not stated	Not stated	Not stated
Ekenna 2020 [52]	Physical presence of drug	Proportion of medicines available	Not stated	Not stated
Fang 2013 [26]	Physical presence of drug	Proportion of facilities where medicine was available	Not stated	Not stated
Fernandopulle 2019 [49]	Patient-reported availability of medicines at health facility	Proportion of individuals reporting medicine was available	Not stated	Not stated
Ibrahim 2020 [32]	Physical presence of drug	Proportion of facilities where each medicine was available	Yes	Not stated
Jigjidsuren 2019 [27]	Not stated	Proportion of medicines available	Not stated	Not stated
Kasonde 2019 [28]	Not stated	Proportion of medicines available	Not stated	Not stated
Katende 2015 [29]	Physical presence of drug	Proportion of facilities where at least one type of medicine was available	Not stated	Yes
Khanal 2019 [30]	Not stated	Proportion of facilities where medicine was available	Not stated	Not stated
Kibridge 2017 [31]	Not stated	Proportion of facilities where medicine was available	Not stated	Not stated
Mendes 2014 [59]	Physical presence of drug	The proportion of health units where greater than 80% of all key drugs from 12 categories were available	Yes	Not stated
Minaei 2019 [32]	Not stated	Proportion of medicines available	Not stated	Not stated
Musinguzi 2015 [33]	Two definitions used, first definition not stated, second definition is presence of reported stock-outs in the prior three months	Proportion of facilities where medicine was available; proportion of facilities reporting stock-outs in prior three months	Not stated	Not stated
Mutale 2018 [34]	Not stated	Proportion of facilities where medicine was available	Not stated	Not stated
Oliveira 2016 [35]	Patient-reported availability of medicines at health facility	Proportion of individuals reporting medicine was available	Not stated	Not stated
Oyekale 2017 [36]	Not stated	Proportion of facilities where medicine was available	Not stated	Yes
Peck 2014 [38]	Physical presence of drug	Proportion of facilities where medicine was available	Not stated	Not stated
Resendez 2000 [58]	Physical presence of drug	Proportion of medicines available; proportion of facilities where zero, 1, 2, or 3 types of medicines were available	Not stated	Not stated
Restinia 2021 [54]	Not stated	Not stated	Not stated	Not stated
Rockers 2019 [40]	Physical presence of drug	Proportion of facilities or households where medicine was available	Yes	Not stated
Rockers 2018 [41]	Physical presence of drug	Proportion of facilities or households where medicine was available	Yes	Not stated
Saeed 2021 [56]	Physical presence of drug	Proportion of facilities where medicine was available	Yes	Not stated
Su 2017 [42]	Physical presence of drug	Proportion of facilities where medicine was available	Not stated	Not stated
Uzuchukwo 2012 [44]	Estimated expected shelf life of a drug	Proportion of medicines available; mean estimated shelf life of a drug across facilities	Not stated	Not stated
Vialle-Valentin 2015 [45]	Physical presence of drug	Proportion of households where medicine was available	Not stated	Not stated
Wirtz 2018 [47]	Physical presence of drug	Proportion of households where at least one type of medicine was available	Yes	Not stated
Yang 2015 [48]	Physical presence of drug	Proportion of medicines available	Not stated	Not stated

**Table 4:** Approaches to measuring affordability used by different studies.

Study	Affordability Definition	Affordability Quantification Approach
Ashighbie 2020 [19]	Ability to obtain medicines for free	Proportion of facilities providing medicines for free
Contreras-Loya 2013 [57]	Ability to obtain medicines for free	Proportion of individuals who obtained medicines for free, proportion of facilities where medicines were available for free
Emmerick 2020 [25]	Paying no co-payment for medicines	Mean monthly co-payments (paying any amount versus zero)
Fernandopulle 2019 [49]	Patient-reported affordability of medicines	Proportion of individuals reporting medicine was affordable
Leao Tavares 2016 [43]	Ability to obtain medicines for free	Proportion of individuals who obtained all of their medicines for free
Paniz 2010 [37]	Ability to obtain medicines for free	Proportion of individuals who obtained medicines for free
Perlman 2011 [39]	Patient-reported affordability of medicines	Proportion of individuals reporting medicine was affordable
Restrepo 2020 [55]	Income impairment as defined by the average amount spent on medicines divided by the family income per capita multiplied by 100	Mean income impairment
Viana 2015 [46]	Ability to obtain medicines for free	Proportion of individuals who obtained all their medicines for free

### Factors associated with availability

#### Individual, household, and community level

Sociodemographic characteristics associated with medicine availability were assessed in four studies, with inconsistent findings [41, 45, 47, 49]. Age was examined in two and was only associated with greater availability in three of five countries in one of the studies [45, 47]. Associations between socioeconomic status and availability varied in the same five-country study, but two other studies found positive associations [41, 45, 47]. Education was also positively associated with availability in one of two studies but not in the other [45, 47]. There was no association between sex and availability of medicines in the three studies looking at this variable [45, 47, 49]. At the community level, seven studies compared medicine availability in urban and rural settings [21, 23, 34, 36, 47, 52, 58], with three reporting no differences [36, 47, 58]. In two studies from Zambia and Kenya that looked at different CVD medicine classes separately, only calcium channel blockers and hydrochlorothiazide had significantly lower availability in rural facilities, respectively [23, 34].

#### Service delivery level

Twenty one studies examined service delivery level arrangements [17, 18, 21–23, 27–29, 31–33, 35, 36, 38, 42, 47, 51, 53, 56, 58, 59]. Thirteen of these compared availability at private and public facilities with mixed results [18, 21–23, 28, 31, 32, 35, 36, 47, 51, 53, 56]. Additionally, 11 studies looked at the relationship between facility tier and medicine availability [17, 18, 21, 27, 29, 33, 36, 38, 42, 58, 59]. In seven studies from Tanzania, Uganda, Nigeria, China, and Brazil, higher tier facilities staffed by medical doctors or providing a wider range of services were significantly more likely to have antihypertensive medicines or a basket of medicines available [18, 21, 29, 33, 36, 42, 59]. Among these, three also looked at correlations between availability and the level of amenities at a facility such as equipment, a pharmacy refrigerator, or solar panels. The presence of such amenities was associated with greater availability of medicines in all three studies [18, 36, 59]. The relationship between service integration and medicine availability was examined in two studies. In Uganda, facilities providing HIV counseling and testing had greater availability of NCD medicines while those offering HIV care had lower availability [18]. In Brazil, primary healthcare units dispensing psychotropic drugs had 3.16 (95% CI: 2.85–3.51) times greater odds of having essential medicines available [59].

#### Health sector (program) level

Eight studies examined health sector level arrangements [20, 26, 28, 35, 42, 44, 54, 56]. Three studies from Brazil, China, and Nigeria all point to an association between facility revenues and medicine availability [26, 35, 44]. For example, in China, the introduction of a policy that eliminated facility mark-ups on drug prices (and thus decreased revenue) was associated with a decrease in mean availability of lowest-priced generics from 25.5% to 20.5% (p < 0.0001) [26]. In a fourth study, from Indonesia, implementation of a national policy that included a shift from fee-for-service to more limited reimbursements based on the type of diagnosis was associated with variable results for different types of antihypertensive agents. This policy also included a requirement that medicines be ordered from the national formulary, however, the study provided little detail on the methodology employed [54].

The relationship between the presence of drugs on essential medicine lists and availability was examined in four studies [20, 26, 28, 56]. In China, the introduction of a provincial essential medicine list was associated with decreases in medicine availability [26]. In Bangladesh, availability of medicines was 50.3% for those on the national essential medicine list and 57% for those not on the list (p > 0.05) [28]. But in a survey of 23 LMICs, median availability of generic medicines on national lists was significantly greater [20]. In Pakistan as well, health facility availability of CVD medicines on the essential medicine list was significantly greater than those not on the list [56].

#### National and international levels

We found no studies assessing the relationship between national policies from outside the health sector and availability. In terms of international factors, one cluster-randomized controlled trial that evaluated a Novartis-sponsored program providing medicines for NCDs in Kenya at $1 per treatment per month found increased availability of amlodipine–but not other CVD medicines–at health facilities [40].

### Factors associated with affordability

#### Individual, household, and community level

Five studies examined sociodemographic factors associated with affordability with four examining socioeconomic status [39, 43, 46, 49, 55]. Two from Brazil revealed a negative association between socioeconomic status and affordability [43, 46], while a third study showed no association [55]. Conversely, a study from Russia revealed a positive association [39].

#### Service delivery level

Three studies examined correlations between affordability and service delivery level indicators [19, 55, 57]. In Kenya, public facilities were more likely to provide free NCD medicines than private facilities (47% v 9%, p<0.0001) [19]. In Mexico, medicines were obtained for free by 62% of those visiting pharmacies that were operated by the state and by 70% of those visiting outsourced pharmacies (p < 0.01) [57]. In Brazil, medicines were more affordable for individuals who reported primarily using public health facilities as part of the Sistemo Unico de Saude to procure medicines than those who used private pharmacies. The Sistemo Unico de Saude is a national program that aims to increase access to medicines by providing certain medications free of charge [55].

#### Health sector (program) level

Four studies examined the association between affordability and the presence of health insurance [39, 43, 46, 55]. In Brazil, not having a health insurance plan was associated with greater medicine affordability [43, 46, 55]. An interrupted time series analysis from Brazil examining a policy removing copayments for antihypertensives found significant reductions in mean out-of-pocket expenditures after the policy was implemented [25].

#### National and international levels

We did not identify any studies that examined factors at the national level (non-health sector) or international level associated with medicine affordability.

## Discussion

To the best of our knowledge, this is the first systematic review of health system factors associated with availability and affordability of CVD medicines in LMICs. Our findings highlight significant gaps in evidence and heterogeneity in results as well as variable quality of existing studies. While certain factors seemed consistently associated with availability, findings regarding other factors were inconsistent.

There are a number of possible reasons for these inconsistencies. First, most studies were cross-sectional and subject to potential confounding, precluding establishment of causal relationships. Intervention studies are needed but the feasibility of implementing them will depend on context, so they should be accompanied by qualitative policy analyses.

Second, availability and affordability of medicines are unlikely to be affected by a single factor. Instead, a multitude of factors must be aligned in complex relationships at different health systems levels [16]. This was evident in several studies, including the sole cluster-randomized trial included, where subsidies from Novartis had little effect on medicine availability in Kenya [40]. This differs from a similar intervention involving subsidies for artemisinin-based combination therapy in sub-Saharan Africa, which achieved notable increases in medicine availability [60]. As the authors of the first study acknowledge, subsidization is only one element in a complex system which also has to account for factors such as physician, pharmacist, and patient awareness of specific medicines, procurement, delivery mechanisms, and inclusion in clinical practice guidelines. This may also explain why the presence of medicines on national essential medicine lists did not translate to greater availability in all included studies. Different countries may go about deciding what medicines to include on these lists differently [20]. It may be that in some contexts, drugs on essential medicine lists are not included in clinical guidelines or are less accepted by prescribers and patients. Other factors may also influence whether medicines on essential lists are more available. One included study demonstrated an inverse relationship between country-income level and availability of medicines on these lists [20]. The authors posit that in countries with fewer resources, essential medicine lists drive prioritization. Interestingly, in two studies we identified from Pakistan [56] and Bangladesh [28], availability of medicines positively correlated with their presence on essential lists only in Pakistan, which has the lower national income level of the two [61]. Similarly, medicines on essential lists tend to be more available at public facilities [20]. Yet, the studies we included analyzed a heterogeneous sample of public and private facilities as well as different tier facilities together. This may explain some of the inconsistencies observed. In a study from China [26], where implementation of an essential medicine list was associated with lower availability, the policy was accompanied by elimination of mark-ups on drug prices at health facilities, which may have reduced revenues for medicine procurement.

We also noted inconsistencies and counterintuitive findings for affordability. In Brazil, the absence of health insurance was associated with greater affordability of medicines [43, 46, 55]. Individuals from lower socioeconomic backgrounds who lack health insurance rely more heavily on Brazilian public health programs like the Sistema Unico de Saude, which provide medicines for free particularly for NCDs. Those with private insurance, on the other hand, are more likely to procure medicines through the private sector where they will be required to pay [62]. Future research must take a more holistic and dynamic approach, looking at how individual correlates of inadequate access are influenced by the characteristics of the health systems in which they operate.

A third possible reason for inconsistent findings is the heterogeneity in measures of availability and affordability. For example, some studies on availability assess the percentage of facilities where a drug is available while others report the proportion of drugs available from a list of medicines. Employing even slightly different measures can yield widely disparate estimates in the same country [63]. This problem is compounded by the fact that most current measures are binary. Many of the included studies considered a medicine available if it was present on the day of the survey regardless of quantity, expiry date, or reliability of supply.

A consistent and comprehensive definition of affordability is also needed. In all but one study, affordability was either ascertained subjectively from respondents or by measuring the ability to obtain medicines for free, which does not fully encapsulate the degree to which medicines are unaffordable for those who have to pay. Many measures of affordability, including those employed by the studies included here, rely on a single measure at one point in time. These measures may not provide a full picture of the long-term financial burden that purchasing medications for chronic conditions places on families [64]. Other metrics of affordability have been described in the literature, however, no study meeting our inclusion criteria used such definitions. For example, the WHO Health Action International Survey measures affordability as the number of days of wages the lowest-paid government worker has to spend on medicines [65]. Many of these approaches involve making arbitrary judgments on thresholds for affordability. Doing so without taking into account households’ disposable income risks overestimating true affordability [13]. The downside of more comprehensive definitions is challenges in measurement. Yet, without them, interventions may be deemed effective if they meet certain predetermined benchmarks, even if they do not truly reflect how available or affordable medicines are for those who need them.

An important finding from our study is the paucity of factors studied that could influence availability and affordability. For example, no study examined the role of corruption, although this has been shown to be negatively associated with access to HIV antiretroviral therapy [66]. Nor were supply chain factors investigated, despite several studies showing how relevant interventions affected availability of medicines for other diseases [67, 68]. Similarly, intellectual property provisions may have implications on availability and affordability of CVD medicines. Different studies have shown that the vast majority of medicines on the WHO Essential Medicine List are not under patent protection [69, 70]. These findings, coupled with extensive evidence that medicines on the essential medicine list remain unavailable and unaffordable in most LMICs, imply that patent provisions are unlikely to be the sole contributor to non-availability and affordability. Nevertheless, patents on newer therapeutics or fixed-dose combination therapies for CVD that are not on essential medicine lists may impact their availability and affordability and warrant further investigation [71].

Our study has some limitations. We restricted our search to factors associated with availability and affordability but did not examine other parameters of access. Though beyond the scope of this review, aspects such as acceptability are also important in ensuring that those who need CVD medicines can receive them [9]. Furthermore, while we did not restrict our search based on language, studies in languages other than English may not be captured in the databases used. Finally, availability and affordability are conceptualized in a multitude of ways. Some studies on this topic may have used terms that were missed by our search terms.

## Conclusion

Evidence concerning factors that influence the availability or affordability of CVD medicines is limited. The majority of studies are observational and while they have identified a number of potential associations, they cannot establish causality. Factors at different levels of the health system likely act together in a multifactorial way to influence availability and affordability of CVD medicines as part of complex health systems. Future research involving uniform definitions and measurement approaches is needed with a particular focus on experimental and quasi-experimental methods that provide insight into causal mechanisms.

## Supporting information

S1 Checklist (DOCX)

S1 Table Complete search terms and search strategy for Medline search. (DOCX)

S2 Table Risk of bias tool for observational studies. (DOCX)

S3 Table Data extraction table. (XLSX)
