# Supplementary material for: Factors associated with the availability and affordability of essential cardiovascular disease medicines in low- and middle-income countries: A systematic review
Source: PLOS Glob Public Health. 2022 Mar 23;2(3):e0000072. doi: 10.1371/journal.pgph.0000072 (PMC10021589; doi:10.1371/journal.pgph.0000072)
Supplement: S1 Table — (DOCX) [file pgph.0000072.s002.docx]

**Appendix I –** Complete search terms for Medline (OVID)

| **Search** | **Topic Theme** | **Search Term** | **Hits** |
| --- | --- | --- | --- |
| 1 | Medicines | exp pharmaceutical preparations/ OR medication*.ti,ab. OR medicines.ti,ab. OR prescription drug*.ti,ab. OR non-prescription drug*.ti,ab. OR nonprescription drug*.ti,ab. OR over-the-counter drug*.ti,ab. OR generic drug*.ti,ab. OR brand-name drug*.ti,ab. OR brandname drug*.ti,ab. OR prescription medicine*.ti,ab. OR non-prescription medicine*.ti,ab. OR nonprescription medicine*.ti,ab. OR over-the-counter medicine*.ti,ab. OR generic medicine*.ti,ab. OR brand-name medicine*.ti,ab. OR brandname medicine*.ti,ab. OR pharmaceutical*.ti,ab. OR pill*.ti,ab. | 1,366,220 |
| 2 | Cardiovascular disease | cardiovascular diseases/ OR exp hypertension/ OR exp hyperlipidemias/ OR cardiovascular disease*.ti,ab. OR CVD.ti,ab. OR CVDs.ti,ab. OR CVD's.ti,ab. OR cardiac disease*.ti,ab. OR heart disease*.ti,ab. OR hyperlipidemia*.ti,ab. OR hyperlipidaemia*.ti,ab. OR hypercholesterolemia*.ti,ab. OR hypercholesterolaemia*.ti,ab. OR dyslipidemia*.ti,ab. OR dyslipidaemia*.ti,ab. OR hypertension.ti,ab. | 944,608 |
| 3 | Non-communicable diseases (NCDs) | exp chronic disease/ OR chronic disease*.ti,ab. OR chronic condition*.ti,ab. OR NCD.ti,ab. OR NCDs.ti,ab. OR NCD's.ti,ab. OR non-communicable disease*.ti,ab. OR noncommunicable disease*.ti,ab. OR non-infectious disease*.ti,ab. OR noninfectious disease*.ti,ab. OR non-transmissible disease*.ti,ab. OR nontransmissible disease*.ti,ab. | 347,498 |
| 4 | Cardiovascular disease drugs | cardiovascular agents/ OR antihypertensive agents/ OR hypolipidemic agents/ OR anticholesterolemic agents/ OR exp platelet aggregation inhibitors/ OR exp angiotensin receptor antagonists/ OR exp angiotensin converting enzyme inhibitors/ OR exp hydroxymethylglutaryl-CoA reductase inhibitors/ OR exp diuretics/ OR exp adrenergic beta-antagonists/ OR exp calcium channel blockers/ OR cardiovascular agent*.ti,ab. OR cardiovascular drug*.ti,ab. OR cardiovascular medicine*.ti,ab. OR cardiovascular medication*.ti,ab. OR cardiovascular disease agent*.ti,ab. OR cardiovascular disease drug*.ti,ab. OR cardiovascular disease medicine*.ti,ab. OR cardiovascular disease medication*.ti,ab. OR antihypertensive*.ti,ab. OR hypertension drug*.ti,ab. OR hypertension medicine*.ti,ab. OR hypertension medication*.ti,ab. OR blood pressure drug*.ti,ab. OR blood pressure medicine*.ti,ab. OR blood pressure medication*.ti,ab. OR blood pressure lowering agent*.ti,ab. OR blood pressure lowering drug*.ti,ab. OR blood pressure lowering medicine*.ti,ab. OR blood pressure lowering medication*.ti,ab. OR hypolipidemic agent*.ti,ab. OR hypolipidemic drug*.ti,ab. OR hypolipidemic medicine*.ti,ab. OR hypolipidemic medication*.ti,ab. OR hypolipidaemic agent*.ti,ab. OR hypolipidaemic drug*.ti,ab. OR hypolipidaemic medicine*.ti,ab. OR hypolipidaemic medication*.ti,ab. OR lipid lowering agent*.ti,ab. OR lipid lowering drug*.ti,ab. OR lipid lowering medicine*.ti,ab. OR lipid lowering medication*.ti,ab. OR hypocholesterolemic agent*.ti,ab. OR hypocholesterolemic drug*.ti,ab. OR hypocholesterolemic medicine*.ti,ab. OR hypocholesterolemic medication*.ti,ab. OR hypocholesterolaemic agent*.ti,ab. OR hypocholesterolaemic drug*.ti,ab. OR hypocholesterolaemic medicine*.ti,ab. OR hypocholesterolaemic medication*.ti,ab. OR antilipidemic agent*.ti,ab. OR antilipidemic medicine*.ti,ab. OR antilipidemic medication*.ti,ab. OR antilipidemic drug*.ti,ab. OR antilipidaemic agent*.ti,ab. OR antilipidaemic medicine*.ti,ab. OR antilipidaemic medication*.ti,ab. OR antilipidaemic drug*.ti,ab. OR antilipemic agent*.ti,ab. OR antilipemic medicine*.ti,ab. OR antilipemic medication*.ti,ab. OR antilipemic drug*.ti,ab. OR antilipaemic agent*.ti,ab. OR antilipaemic medicine*.ti,ab. OR antilipaemic medication*.ti,ab. OR antilipaemic drug*.ti,ab. OR anticholesterolemic agent*.ti,ab. OR anticholesterolemic medicine*.ti,ab. OR anticholesterolemic medication*.ti,ab. OR anticholesterolemic drug*.ti,ab. OR anticholesterolaemic agent*.ti,ab. OR anticholesterolaemic medicine*.ti,ab. OR anticholesterolaemic medication*.ti,ab. OR anticholesterolaemic drug*.ti,ab. OR hypolipemic agent*.ti,ab. OR hypolipemic medicine*.ti,ab. OR hypolipemic medication*.ti,ab. OR hypolipemic drug*.ti,ab. OR hypolipaemic agent*.ti,ab. OR hypolipaemic medicine*.ti,ab. OR hypolipaemic medication*.ti,ab. OR hypolipaemic drug*.ti,ab. OR cholesterol agent*.ti,ab. OR cholesterol drug*.ti,ab. OR cholesterol medicine*.ti,ab. OR cholesterol medication*.ti,ab. OR antiplatelet agent*.ti,ab. OR antiplatelet drug*.ti,ab. OR antiplatelet medicine*.ti,ab. OR antiplatelet medication*.ti,ab. OR anti-platelet agent*.ti,ab. OR anti-platelet drug*.ti,ab. OR anti-platelet medicine*.ti,ab. OR anti-platelet medication*.ti,ab. OR platelet aggregation inhibitor*.ti,ab. OR aspirin.ti,ab. OR angiotensin receptor antagonist*.ti,ab. OR angiotensin receptor blocker*.ti,ab. OR angiotensin converting enzyme inhibitor*.ti,ab. OR dipeptidyl carboxypeptidase inhibitor*.ti,ab. OR hydroxymethylglutaryl-CoA reductase inhibitor*.ti,ab. OR HMG-CoA reductase inhibitor*.ti,ab. OR statin*.ti,ab. OR diuretic*.ti,ab. OR beta-blocker*.ti,ab. OR adrenergic beta-antagonist*.ti,ab. OR calcium channel blocker*.ti,ab. | 604,667 |
| 5 | Availability and affordability | Drug costs/ OR exp fees, pharmaceutical/ OR affordab*.ti,ab. OR cost*.ti,ab. OR price*.ti,ab. OR availab*.ti,ab. OR access*.ti,ab. OR obtainab*.ti,ab. OR supply.ti,ab. OR supplies.ti,ab. OR stock*.ti,ab. OR presen*.ti,ab. | 7,763,817 |
| 6 | LMICs | ((developing OR less* developed OR under developed OR underdeveloped OR middle income OR low* income OR underserved OR under served OR deprived OR poor*) adj (economy OR economies)).ti,ab. OR ((developing OR less* developed OR under developed OR underdeveloped OR middle income OR low* income OR underserved OR under served OR deprived OR poor*) adj (countr* OR nation? OR population? OR world)).ti,ab. OR (low* adj (gdp OR gnp OR gross domestic OR gross national)).ti,ab. OR (low adj3 middle adj3 countr*).ti,ab. OR (lmic OR lmics OR third world OR lami countr*).ti,ab. OR transitional countr*.ti,ab. OR global south.ti,ab. OR developing countries/ OR exp africa south of the sahara/ OR ("africa south of the sahara" OR sub-saharan africa OR central africa OR eastern africa OR southern africa OR western africa).ti,ab. OR "Democratic People's Republic of Korea"/ OR (north korea OR (democratic people* republic adj2 korea)).ti,ab. OR Cambodia/ OR cambodia.ti,ab. OR Indonesia/ OR indonesia.ti,ab. OR Micronesia/ OR Kiribati.ti,ab. OR Laos/ OR (laos OR (lao adj1 democratic republic)).ti,ab. OR (marshall island* OR caroline island* OR ellice island* OR gilbert island* OR johnston island* OR mariana island* OR micronesia OR pacific island*).ti,ab. OR Mongolia/ OR mongolia.ti,ab. OR Myanmar/ OR (myanmar OR burma).ti,ab. OR Papua New Guinea/ OR Papua New Guinea.ti,ab. OR Philippines/ OR Philippines.ti,ab. OR Timor-Leste/ OR Timor-Leste.ti,ab. OR Vanuatu/ OR Vanuatu.ti,ab. OR Vietnam/ OR (Viet Nam OR Vietnam).ti,ab. OR American Samoa/ OR american samoa.ti,ab. OR exp China/ OR china.ti,ab. OR Fiji/ OR fiji.ti,ab. OR Malaysia/ OR malaysia.ti,ab. OR marshall islands.ti,ab. OR nauru.ti,ab. OR samoa/ OR "independent state of samoa"/ OR ("independent state of samoa" OR (samoa NOT american samoa) OR western samoa OR navigator islands OR samoan islands).ti,ab. OR Thailand/ OR Thailand.ti,ab. OR Tonga/ OR tonga.ti,ab. OR Tuvalu.ti,ab. OR Armenia/ OR Armenia.ti,ab. OR "Georgia (Republic)"/ OR Kosovo/ OR Kosovo.ti,ab. OR Kyrgyzstan/ OR (kyrgyzstan OR kyrgyz republic OR kirghizia OR kirghiz).ti,ab. OR Moldova/ OR Moldova.ti,ab. OR Tajikistan/ OR tajikistan.ti,ab. OR Ukraine/ OR Ukraine.ti,ab. OR Uzbekistan/ OR Uzbekistan.ti,ab. OR Albania/ OR Albania.ti,ab. OR Azerbaijan/ OR Azerbaijan.ti,ab. OR "Republic of Belarus"/ OR (belarus OR byelarus OR belorussia).ti,ab. OR Bosnia-Herzegovina/ OR (bosnia OR herzegovina).ti,ab. OR Bulgaria/ OR Bulgaria.ti,ab. OR Kazakhstan/ OR (Kazakhstan OR kazakh).ti,ab. OR "Macedonia (Republic)"/ OR Macedonia.ti,ab. OR Montenegro/ OR Montenegro.ti,ab. OR Romania/ OR Romania.ti,ab. OR exp Russia/ OR USSR/ OR (Russia OR Russian Federation OR USSR OR Union of Soviet Socialist Republics OR Soviet Union).ti,ab. OR Serbia/ OR serbia.ti,ab. OR Turkey/ OR (turkey.ti,ab. NOT animal/) OR Turkmenistan/ OR Turkmenistan.ti,ab. OR Yugoslavia/ OR yugoslavia.ti,ab. OR Haiti/ OR Haiti.ti,ab. OR Bolivia/ OR Bolivia.ti,ab. OR El Salvador/ OR El Salvador.ti,ab. OR Guatemala/ OR Guatemala.ti,ab. OR Honduras/ OR Honduras.ti,ab. OR Nicaragua/ OR Nicaragua.ti,ab. OR Belize/ OR Belize.ti,ab. OR Brazil/ OR Brazil.ti,ab. OR Argentina/ OR Argentina.ti,ab. OR Colombia/ OR Colombia.ti,ab. OR Costa Rica/ OR Costa Rica.ti,ab. OR Cuba/ OR Cuba.ti,ab. OR Dominica/ OR Dominica.ti,ab. OR Dominican Republic/ OR Dominican Republic.ti,ab. OR Ecuador/ OR Ecuador.ti,ab. OR Grenada/ OR Grenada.ti,ab. OR Guyana/ OR Guyana.ti,ab. OR Jamaica/ OR Jamaica.ti,ab. OR Mexico/ OR Mexico.ti,ab. OR Paraguay/ OR Paraguay.ti,ab. OR Peru/ OR Peru.ti,ab. OR Saint Lucia/ OR (St Lucia OR Saint Lucia).ti,ab. OR "Saint Vincent and the Grenadines"/ OR Grenadines.ti,ab. OR Suriname/ OR Suriname.ti,ab. OR Venezuela/ OR Venezuela.ti,ab. OR Djibouti/ OR (Djibouti OR French Somaliland).ti,ab. OR Egypt/ OR Egypt.ti,ab. OR Jordan/ OR Jordan.ti,ab. OR Morocco/ OR Morocco.ti,ab. OR Syria/ OR (Syria OR Syrian Arab Republic).ti,ab. OR Tunisia/ OR tunisia.ti,ab. OR Gaza.ti,ab. OR Yemen/ OR Yemen.ti,ab. OR Algeria/ OR Algeria.ti,ab. OR Iran/ OR Iran.ti,ab. OR Iraq/ OR Iraq.ti,ab. OR Jordan/ OR Jordan.ti,ab. OR Lebanon/ OR Lebanon.ti,ab. OR Libya/ OR Libya.ti,ab. OR Afghanistan/ OR Afghanistan.ti,ab. OR Nepal/ OR Nepal.ti,ab. OR Bangladesh/ OR Bangladesh.ti,ab. OR Bhutan/ OR Bhutan.ti,ab. OR exp India/ OR India.ti,ab. OR Pakistan/ OR Pakistan.ti,ab. OR Sri Lanka/ OR Sri Lanka.ti,ab. OR Indian Ocean Islands/ OR Maldives.ti,ab. OR Benin/ OR (Benin OR Dahomey).ti,ab. OR Burkina Faso/ OR (Burkina Faso OR Burkina Fasso OR Upper Volta).ti,ab. OR Burundi/ OR Burundi.ti,ab. OR Central African Republic/ OR (Central African Republic OR Ubangi-Shari).ti,ab. OR Chad/ OR Chad.ti,ab. OR Comoros/ OR (Comoros OR Comoro Islands OR Mayotte OR Iles Comores).ti,ab. OR "Democratic Republic of the Congo"/ OR ((democratic republic adj2 congo) OR belgian congo OR zaire).ti,ab. OR Eritrea/ OR Eritrea.ti,ab. OR Ethiopia/ OR Ethiopia.ti,ab. OR Gambia/ OR Gambia.ti,ab. OR Guinea/ OR (Guinea NOT (New Guinea OR Guinea Pig* OR Guinea Fowl)).ti,ab. OR Guinea-Bissau/ OR (Guinea-Bissau OR Portuguese Guinea).ti,ab. OR Liberia/ OR Liberia.ti,ab. OR Madagascar/ OR (Madagascar or Malagasy Republic).ti,ab. OR Malawi/ OR (Malawi OR Nyasaland).ti,ab. OR Mali/ OR Mali.ti,ab. OR Mozambique/ OR (Mozambique OR Mocambique OR Portuguese East Africa).ti,ab. OR Niger/ OR (Niger NOT (Aspergillus OR Peptococcus OR Schizothorax OR Cruciferae OR Gobius OR Lasius OR Agelastes OR Melanosuchus OR radish OR Parastromateus OR Orius OR Aspergillus OR Parastromateus OR Stomoxys)).ti,ab. OR Rwanda/ OR (Rwanda OR Ruanda).ti,ab. OR Senegal/ OR senegal.ti,ab. OR Sierra Leone/ OR Sierra Leone.ti,ab. OR Somalia/ OR Somalia.ti,ab. OR South Sudan/ OR south sudan.ti,ab. OR Tanzania/ OR (Tanzania OR Tanganyika OR Zanzibar).ti,ab. OR Togo/ OR (Togo OR Togolese Republic).ti,ab. OR Uganda/ OR Uganda.ti,ab. OR Zimbabwe/ OR (Zimbabwe OR Rhodesia).ti,ab. OR Angola/ OR angola.ti,ab. OR Cameroon/ OR Cameroon.ti,ab. OR Cape Verde/ OR (Cape Verde OR Cabo Verde).ti,ab. OR Congo/ OR (congo NOT ((democratic republic adj3 congo) OR congo red OR crimean-congo)).ti,ab. OR Cote d'Ivoire/ OR (Cote d'Ivoire OR Ivory Coast).ti,ab. OR Ghana/ OR (Ghana OR Gold Coast).ti,ab. OR Kenya/ OR kenya.ti,ab. OR Lesotho/ OR (Lesotho OR Basutoland).ti,ab. OR Mauritania/ OR Mauritania.ti,ab. OR Nigeria/ OR Nigeria.ti,ab. OR Atlantic Islands/ OR (sao tome adj2 principe).ti,ab. OR Sudan/ OR (Sudan NOT South Sudan).ti,ab. OR Swaziland/ OR (Swaziland OR Eswatini).ti,ab. OR Zambia/ OR (Zambia OR Northern Rhodesia).ti,ab. OR Botswana/ OR (Botswana OR Bechuanaland OR Kalahari).ti,ab. OR Equatorial Guinea/ OR (Equatorial Guinea OR Spanish Guinea).ti,ab. OR Gabon/ OR (Gabon OR Gabonese Republic).ti,ab. OR Mauritius/ OR (Mauritius OR Agalega Islands).ti,ab. OR Namibia/ OR Namibia.ti,ab. OR South Africa/ OR South Africa.ti,ab. | 1,617,866 |
| 7 | Cardiovascular disease AND drugs | 1 AND 2 | 68,351 |
| 8 | NCDs AND drugs | 1 AND 3 | 24,275 |
| 9 | All drugs | 4 OR 7 OR 8 | 661,644 |
| 10 | All drugs AND availability/affordability AND LMICs | 9 AND 5 AND 6 | 6,840 |
| 11 | Filters | Published after 2000 | 6,376 |
