# Supplementary material for: Factors associated with the availability and affordability of essential cardiovascular disease medicines in low- and middle-income countries: A systematic review
Source: PLOS Glob Public Health. 2022 Mar 23;2(3):e0000072. doi: 10.1371/journal.pgph.0000072 (PMC10021589; doi:10.1371/journal.pgph.0000072)
Supplement: S2 Table — (DOCX) [file pgph.0000072.s003.docx]

**Appendix II -** Tool for assessing risk of bias for observational studies

| **Type of bias** | **Study design** | | | |
| --- | --- | --- | --- | --- |
|  | **Cross sectional** | **Case control** | **Cohort** | **Ecological** |
| **Selection bias** | Was the study population selected appropriate? | | | |
|  | Was the sample representative of its target population? | Were the controls randomly selected from the same population as the cases? | Was an appropriate control group used?  Was follow up sufficiently complete? (>80%) | Were the subjects representative of the group, place, or population of interest? |
| **Differential misclassification** | Did the assessment of the exposure or outcome differ in different groups? | Did the exposure assessment differ for cases and controls? | Did the outcome assessment differ for exposed and non exposed? | Were the exposure and outcome variables measured and defined in the same or a similar way across the different groups studied? |
| **Non-differential misclassification** | Were valid methods used for measuring medicine availability and/or affordability? | | | |
| **Confounding** | Was any strategy undertaken to control for potential confounders?   1. At the design stage (restriction, matching) 2. At the analysis stage (stratification, multivariable analysis) | | | |

**Define each domain as low risk of bias, unclear risk of bias or high risk of bias**
